# Supplementary material for: Exploring the toxicological mechanisms of Benzo[a]anthracene (BaA) exposure in lung adenocarcinoma (LUAD) via network toxicology, machine learning, and multi-dimensional bioinformatics analysis
Source: PLoS One. 2026 Feb 11;21(2):e0340116. doi: 10.1371/journal.pone.0340116 (PMC12893569; doi:10.1371/journal.pone.0340116)
Supplement: Attachment — Submitted filename: Response to Reviewers.docx [file pone.0340116.s005.docx]

**Response to Reviewers**

Dear Editor and Reviewers,

Thank you for the opportunity to revise our manuscript entitled "Exploring the toxicological mechanisms of Benzo [a] anthracene (BaA) exposure in lung adenocarcinoma via network toxicology, machine learning, and multi-dimensional bioinformatics analysis" (Manuscript ID: PONE-D-25-55154). We sincerely appreciate the valuable comments and suggestions from all reviewers, which have helped us significantly improve the quality of our work.

We have carefully addressed all points raised in the review process. Below, we provide a point-by-point response to each comment. All corresponding changes have been incorporated into the revised manuscript using the "Track Changes" function.

We believe that the criticisms have been addressed effectively and that the revisions have substantially strengthened the manuscript. We are grateful for the guidance provided and hope that the revised version now meets the high publication standards of PLOS ONE.

Thank you again for considering our work. We look forward to hearing from you.

Sincerely,

Zhiyao Shi, Xixing Wang
On behalf of all co-authors

**Response to Editorial Requirements：**

Dear Dr. Jiafu Li and Editorial Team,

Thank you for the opportunity to revise our manuscript. We have carefully addressed all the points raised by the reviewers and have meticulously ensured that our manuscript now fully complies with PLOS ONE's journal requirements. Our point-by-point responses to the reviewers are detailed in the separate "Response to Reviewers" document.

Regarding the specific journal formatting and policy requirements, we have taken the following actions:

Style Requirements: We have reformatted the entire manuscript to ensure it strictly adheres to PLOS ONE's style templates.

ORCID iD: The corresponding author's ORCID iD has been validated in the Editorial Manager system.

Code Sharing: In full compliance with PLOS ONE's policy on code sharing, we have deposited all author-generated R and Python scripts for statistical analysis and visualization, along with the parameter files for molecular docking and dynamics simulations (including session information and software versions), to the Zenodo repository. The dataset is publicly accessible under the DOI: [https://doi.org/10.5281/zenodo.17747122](https://doi.org/10.5281/zenodo.17747122). The Data Availability Statement in the manuscript has been updated accordingly.

Ethics Statement: The ethics statement has been moved exclusively to the Methods section (Section 2.10) and removed from any other part of the manuscript.

Funding Statement: The following sentence has been added to the manuscript to clarify the funder's role: "The funders had no role in study design, data collection and analysis, decision to publish, or preparation of the manuscript." We request that the online submission form's Funding Statement be updated to match the one provided in the manuscript and cover letter.

Acknowledgments: All funding-related text has been removed from the Acknowledgments section of the manuscript. The Acknowledgments now only contain non-financial contributions. The funding information is now presented solely in the Funding Statement section.

Abstract Consistency: We have verified that the abstract in the manuscript and the one on the online submission form are identical.

Figure References: We have thoroughly checked the entire manuscript and confirmed that all figures (1 through 8) are appropriately referenced in the text.

Figure Quality: New, high-resolution copies of Figures 1, 2, 3, and 4 have been uploaded as per the journal's guidelines for figure clarity.

Suggested Citations: We have reviewed the publications suggested by the reviewers. Where we found the suggested literature to be relevant and to strengthen the scholarly context of our work, we have incorporated the citations. All new citations have been integrated appropriately.

We believe that the manuscript has been significantly improved through the revision process and now meets all the standards of PLOS ONE. Thank you for your time and consideration.

Sincerely,

Zhiyao Shi, Xixing Wang
On behalf of all co-authors

**Response to Reviewer #1:**

**Dear Reviewer #1,**

We sincerely appreciate your meticulous review and valuable insights on our manuscript. Your professional comments have provided crucial guidance for improving the quality and rigor of our study, and we fully agree with all the revision suggestions you proposed. We have carefully addressed each comment and made corresponding revisions to the manuscript. Below is a detailed response to each of your points:

**Comment 1:** Lack of In Vitro/In Vivo Functional Validation: Core conclusions rely on bioinformatics and computational simulations without validation via in vitro cell experiments or in vivo models; suggest supplementing in vitro experiments for 1–2 key targets.

**Response:** First and foremost, we fully concur with your insightful suggestion that in vitro/in vivo functional validation is essential to strengthen the reliability of our core conclusions. We deeply recognize that experimental verification would greatly enhance the translational value and persuasiveness of our findings. However, due to several unavoidable practical constraints, we were unable to conduct such experiments in the current study. Specifically, these constraints include: (1) limitations in sample availability, as obtaining high-purity BaA standard samples and matched LUAD cell models requires strict quality control and specialized resources; (2) restrictions in laboratory facilities, as our research team’s current setup lacks dedicated cell culture platforms, biosafety cabinets, and other equipment necessary for in vitro toxicology experiments; (3) technical and equipment barriers related to BaA extraction and purification, which demands advanced chromatographic systems and professional operational skills that are not available in our laboratory; and (4) limited research funding, which cannot cover the high costs of reagent procurement, cell line maintenance, animal ethics approval, and specialized technical training.

To mitigate this limitation and improve the robustness of our conclusions, we have implemented the following revisions: (1) We further refined the screening criteria for BaA-related targets, excluding pan-genes and low-confidence targets to focus on those with direct biological relevance to BaA-induced LUAD progression; (2) We enhanced the depth of existing data analysis (3) We supplemented 5 recent high-quality studies (cited in the revised manuscript) that have validated the functional roles of key targets (e.g., CRABP2 and CXCL12) in LUAD through in vitro/in vivo experiments, providing indirect experimental support for our computational predictions; (4) We explicitly acknowledged this limitation in the "Limitations and Future Perspectives" section and discussed its potential impact on the interpretation of our results. In future research, we aim to improve our experimental conditions, seek collaborations with leading domestic laboratories specializing in environmental toxicology and oncology, and conduct in vitro experiments (e.g., target overexpression/knockdown assays combined with cell proliferation, migration, and invasion tests) to directly validate the regulatory roles of core targets in BaA-promoted LUAD progression.

**Comment 2:** Single Dataset Limitation: Core target validation relies solely on GSE10072 (sample size unspecified); suggest adding 1–2 independent datasets for validation and specifying GSE10072’s sample size.

**Response:** We highly appreciate your reminder regarding the limitation of relying on a single dataset. To address this issue, we have made the following revisions: (1) We supplemented two additional independent LUAD transcriptomic datasets from the GEO database, namely GSE32863 (58 LUAD tissues and 58 matched adjacent normal tissues) and GSE31210 (226 LUAD tissues and 20 normal lung tissues); (2) We specified the sample size of GSE10072 in the revised manuscript: this dataset includes 58 LUAD tissue samples and 49 normal lung tissue samples; (3) We integrated the three datasets, removed batch effects, and performed unified standardization and differential expression analysis. The validation results consistently show that the five core targets (TNNC1, ABCC3, CRABP2, CXCL12, OLR1) exhibit significant differential expression between LUAD and normal tissues across all three datasets (p < 0.01), with AUC values ranging from 0.841 to 0.886, confirming the stability and reliability of our core target identification. The integrated analysis results have been updated in Section 3.4 and the corresponding figures.

**Comment 3:** Ignoring Pollutant Synergistic Effects: BaA’s coexistence with other pollutants and their synergistic carcinogenicity are unaddressed; suggest expanding the "Discussion" and "Limitation" section to discuss or analyze interactions if relevant data exist.

**Response:** We agree with your important point that the synergistic carcinogenic effects of BaA with other coexisting pollutants are a critical aspect of environmental toxicology research. In response, we have expanded the "Limitations and Future Perspectives" section (Section 4.6) . Specifically, we discussed that in real environmental scenarios, BaA often coexists with other pollutants such as polycyclic aromatic hydrocarbons (PAHs, e.g., benzo[a]pyrene), heavy metals (e.g., cadmium), and volatile organic compounds (VOCs). These pollutants may exert synergistic carcinogenic effects by amplifying oxidative stress, enhancing DNA adduct formation, synergistically activating oncogenic pathways (e.g., PI3K-AKT and MAPK), and jointly remodeling the tumor immune microenvironment. We also cited relevant studies to support these potential synergistic mechanisms. Due to the lack of available data on BaA-pollutant mixture exposure and LUAD progression, we were unable to conduct quantitative analysis, but we have highlighted this as a key direction for future research, suggesting that subsequent studies could explore synergistic effects through in vitro mixed exposure experiments and large-scale epidemiological cohort studies.

**Comment 4:** Incomplete Figure Details: Some figures lack key details (e.g., Venn diagram target counts, GO/KEGG plot FDR values, molecular docking critical amino acid residues); suggest supplementing these to let readers obtain core data directly from figures.

**Response:** Thank you for pointing out the incompleteness of figure details. We have carefully revised the relevant figures to supplement all missing key information: (1) For the Venn diagrams (Figure 1), we added specific target counts for each intersection and non-intersection region to enable readers to directly obtain the number of targets identified from each database and their overlaps; (2) For the GO/KEGG enrichment analysis plots (Figure 2), we supplemented the FDR values for each enriched term and labeled them on the corresponding bars/points, allowing readers to assess the statistical significance of the enrichment results directly from the figures; (3) For the molecular docking diagrams (Figure 6), we labeled the critical amino acid residues involved in the binding between BaA and each core target. We have also updated the figure legends to describe these supplementary details clearly. All revised figures now enable readers to obtain core data and key information directly, improving the transparency and readability of the study.

**Comment 5:** Unified Terminology: Inconsistent abbreviations (e.g., "Benzo[a]anthracene" vs. "BaA", "epithelial-mesenchymal transition" vs. "EMT") reduce readability; suggest defining abbreviations on first use (e.g., "Benzo[a]anthracene (BaA)") and standardizing usage.

**Response:** We apologize for the inconsistent use of abbreviations in the original manuscript. We have thoroughly checked the entire manuscript and made comprehensive revisions to unify the terminology: (1) All abbreviations are defined with their full names when first mentioned (e.g., "Benzo[a]anthracene (BaA)", "epithelial-mesenchymal transition (EMT)", "single-sample gene set enrichment analysis (ssGSEA)"); (2) We standardized the use of abbreviations throughout the manuscript, ensuring that the same abbreviation is used consistently after the initial definition; (3) We created a detailed "Abbreviations" section at the beginning of the manuscript to list all abbreviations and their corresponding full names for easy reference. These revisions have significantly improved the readability and consistency of the manuscript.

Once again, we would like to express our sincere gratitude for your valuable time and professional guidance. We believe that the revisions made in response to your comments have greatly improved the quality, rigor, and completeness of our manuscript. We hope that the revised version meets PLOS ONE’s publication standards. Please do not hesitate to contact us if you have any further questions or require additional revisions.

Sincerely,

The Authors

**Response to Reviewer #2:**

**Dear Reviewer #2,**

We sincerely thank the reviewer for their positive evaluation of our work and for their valuable and constructive comments, which have helped us further improve the manuscript. We have addressed all points raised in detail below.

**Comment 1**: Unify chemical nomenclature to “benzo[a]anthracene (BaA)” across title and Methods. Correct “BPS” to “BaA” in Section 2.1 and keep PubChem CID consistent throughout. Also fix the Fig. 5 caption (“five core targets”) to “seven”.

**Response:** We sincerely thank the reviewer for this meticulous observation. As suggested, we have now uniformly used the nomenclature "benzo[a]anthracene (BaA)" upon its first mention in the title, abstract, and throughout the Methods section. The accidental use of "BPS" in Section 2.1 has been corrected to "BaA," and the PubChem CID has been verified for consistency across the manuscript. Furthermore, following a more refined screening process for BaA-related targets, the final number of identified core targets is indeed five. Consequently, we have updated the caption of Fig. 5 (and all related instances in the text) from "seven" to "five core targets" to accurately reflect our findings.

**Comment 2:** PLOS ONE requires open access to underlying data and analysis scripts. “Available upon reasonable request” is not compliant. Please deposit all R/Python scripts and parameter files (with session info/versions) to an open repository (e.g., Zenodo/OSF; GitHub mirrored to Zenodo) and provide a DOI in the Data Availability Statement.

**Response:** We thank the reviewer for highlighting this important requirement. In full compliance with PLOS ONE's data policy, we have now deposited all relevant R and Python scripts for statistical analysis and visualization, along with the parameter files used for molecular docking and dynamics simulations, including session information and software versions, to the Zenodo repository. The dataset is publicly accessible and has been assigned a DOI: https://doi.org/10.5281/zenodo.17747122. The Data Availability Statement in the manuscript has been updated accordingly to provide this direct link.

**Comment 3:** Please add full reproducibility details for docking/MD: PDB IDs/chains and preprocessing; Vina grid center/size, exhaustiveness, replicates, scoring/version; MD time step, constraints, cutoffs, thermostat/barostat, trajectory interval, and exact equilibration/production settings; plus random seeds and software versions. The current text lists tools but lacks parameters.

**Response:** We appreciate the reviewer's valuable suggestion to enhance the reproducibility of our computational experiments. As requested, we have now comprehensively detailed all key parameters for both the molecular docking and molecular dynamics (MD) simulations in the revised Methods sections (Sections 2.6 and 2.7) and corresponding Results sections (3.6 and 3.7). This includes specific PDB IDs and chains used, preprocessing steps, AutoDock Vina grid parameters (center, size, exhaustiveness), number of replicates, scoring function version, MD parameters (time step, constraints, cutoffs, thermostat/barostat settings, trajectory saving frequency), precise descriptions of the equilibration and production phases, random seeds, and explicit software versions.

**Comment 4:** Given the small size of GSE10072 and near-perfect single-gene AUCs, report sample sizes and provide ROC 95%CIs with resampling. Add independent validation cohorts (e.g., TCGA-LUAD, GSE31210/GSE72094) and consider multi-marker models with calibration/decision-curve analysis to mitigate overfitting. Document these steps in Methods/Results.

**Response:** We are grateful for the reviewer's insightful suggestion, which significantly strengthens the validation of our findings. To address the concern regarding dataset size and potential overfitting, we have taken the following actions:

1. The sample sizes for all datasets used are now explicitly reported.

2. We have supplemented the validation of the core targets by incorporating two additional independent cohorts, GSE32863 and GSE31210, alongside the original GSE10072.

3. As suggested, we now report the 95% confidence intervals (CIs) for the ROC curves, calculated using resampling methods (bootstrap).

4. Furthermore, we have developed a multi-marker model based on the core targets and performed both calibration curve analysis and decision-curve analysis to rigorously evaluate the model's clinical utility and further mitigate overfitting concerns.

All these additional analyses and their detailed methodologies have been thoroughly documented in the revised Methods section (Section 2.5) and the corresponding Results section (Section 3.4).

**Comment 5:** To enhance the connection between the BaA (PAH) chemical background, ROS/¹O₂ related discussions and methodologies, it is recommended to supplement the following literature in the introduction/discussion: 10.1002/cjoc.202300637; 10.1002/cjoc.202400602; 10.1002/cjoc.202300614.

**Response:** We thank the reviewer for this excellent suggestion to better contextualize the chemical background of BaA and its link to reactive oxygen species (ROS) and singlet oxygen (¹O₂). Following a careful review of the recommended literature, we have integrated the highly relevant work (10.1002/cjoc.202300637), which discusses PAH-related oxidative stress mechanisms, into the Introduction section to strengthen the foundational background. However, after thorough examination, we found that the specific focus of the other two suggested papers (10.1002/cjoc.202400602: Visible Light-Promoted Aerobic Oxidation of α-Silyl Styrenes with Alcohols and 10.1002/cjoc.202300614: Heteroatom-Doped Graphene Nanoribbons: Precision Synthesis and Emerging Properties) falls outside the direct scope and central narrative of our current study, which focuses on the toxicological mechanisms of BaA in a biological system. Therefore, to maintain focus and relevance, we have cited the former paper where appropriate. We hope this selective incorporation meets with the reviewer's understanding.

Once again, we would like to express our sincere gratitude for your valuable time and professional guidance. We believe that the revisions made in response to your comments have greatly improved the quality, rigor, and completeness of our manuscript. We hope that the revised version meets PLOS ONE’s publication standards. Please do not hesitate to contact us if you have any further questions or require additional revisions.

Sincerely,

The Authors

**Response to Reviewer #3:**

**Dear Reviewer #3,**

We are deeply grateful to the reviewer for their thorough assessment and for raising these critical points. Their meticulous observations have been invaluable in identifying inconsistencies that have significantly improved the rigor and clarity of our manuscript. We have addressed each comment with the utmost care, as detailed below.

**Comment 1:** In the manuscript, there is a problem with the inconsistent use of abbreviations. Abbreviations such as LUAD, BaA, PAHs, BPS, and AOP are either redefined multiple times after the first definition or used without any definition.

**Response:** We sincerely apologize for the inconsistencies in abbreviation usage in our original manuscript. Thank you for bringing this to our attention. We have now conducted a comprehensive review and implemented a systematic revision to standardize terminology throughout the text:

1. All abbreviations are now clearly defined upon their first use in the main text (e.g., "benzo[a]anthracene (BaA)", "epithelial-mesenchymal transition (EMT)", "single-sample gene set enrichment analysis (ssGSEA)").

2. We have ensured that the abbreviated form is used consistently thereafter across all sections.

3. A new "Abbreviations" section has been added at the end of the manuscript, providing a complete list of all abbreviations and their full terms for easy reference.

We believe these changes have substantially enhanced the readability and terminological consistency of the manuscript.

**Comment 2:** In section 3.2 “Functional enrichment analysis of BaA-induced LUAD,” there are obvious writing errors. For example, in phrases like "gland development," "epithelial cell proliferation," the quotation marks are misplaced. The comma should be placed outside the quotation marks.

**Response:** We thank the reviewer for this precise correction regarding punctuation formatting. We have carefully reviewed the entire manuscript and corrected all instances of misplaced punctuation in relation to quotation marks, including those in Section 3.2, to adhere to standard English typographical conventions.

**Comment 3:** In section 2.4 “Screening of Core Targets by Machine Learning,” the authors mention using two machine learning models (both classification-based models) to identify core targets. However, it is unclear how the models determine which genes are the core targets and on what basis. This section should include a detailed description of how the training data were prepared.

**Response:** We thank the reviewer for this crucial comment, which has helped us greatly improve the transparency of our methodology. In response, we have substantially revised Section 2.4. Firstly, we refined the screening criteria for BaA-related targets to exclude pan-genes and low-confidence targets. Secondly, we identified core target genes from 248 BaA-induced LUAD-related targets using lasso, SVM-RFE, and random forest algorithms. The corresponding content has been explained in the text.

**Comment 4:** The gene name formatting also seems problematic. I believe the gene symbols should be italicized, as they represent genes rather than proteins.

**Response:** We appreciate the reviewer's attention to this important detail. The gene symbols throughout the manuscript, in all figure captions, and in the main text have now been formatted in italics to correctly represent genes, in accordance with standard genetic nomenclature.

**Comment 5:** In section 3.5 “Verify the expression and diagnostic effectiveness of seven key targets in lung adenocarcinoma,” Figure 5B shows extremely high (almost perfect) AUC values, such as 0.998 or 0.996, which I believe are classic warning signs of overfitting. It is unclear on which dataset these ROC curves (Figure 5B) were plotted.

**Response:** We agree completely with the reviewer that extremely high AUC values can be a red flag for overfitting and thank them for prompting us to address this more rigorously. To mitigate this concern and enhance the validation of our findings, we have taken the following actions, which are now detailed in the revised Methods (Section 2.5) and Results (Section 3.4):

1. We have supplemented the validation by incorporating two additional independent cohorts (GSE32863 and GSE31210) alongside the original GSE10072 dataset. The specific dataset used for each analysis is now explicitly stated in the figure legends and text.

2. We now report the 95% confidence intervals for all ROC curves, calculated using bootstrap resampling methods.

3. Furthermore, we developed a multi-marker model based on the core targets and performed calibration curve and decision-curve analyses to rigorously evaluate the model's clinical utility and further address potential overfitting. The exceptionally high AUC values originally reported in a single dataset have been contextualized within this more comprehensive validation framework.

**Comment 6:** In section 3.5 “Verify the expression and diagnostic effectiveness of seven key targets in lung adenocarcinoma,” the authors state that "Compared with normal tissues, lung adenocarcinoma lesions showed significantly elevated expression of FABP4, FOXF1, MMRN2, NME1, CD34, LYVE1, and SULF1 (Fig. 5A)." However, upon examining Figure 5A closely, I found that only NME1 and SULF1 are upregulated, while the others are downregulated. I believe this major inconsistency between the text and data (Figure 5A) critically undermines the validity of the subsequent analyses...

**Response:** We are profoundly grateful to the reviewer for identifying this critical discrepancy between our initial textual description and the actual data presented in the figure (now Fig. 4C). This was a significant oversight on our part, and we sincerely apologize for the error and the confusion it caused. Following a refinement of the BaA-related target screening criteria and the application of three machine learning methods, the final set of core targets has been revised to five. We have meticulously re-examined the expression data for these five core targets in the updated figures (Fig. 4C and Fig. 5A-B) and have corrected all subsequent textual descriptions in the manuscript to accurately reflect the true direction of expression change (up- or down-regulation) for each specific gene.

This correction has had a cascading and vital impact on the interpretation of our results, and we have therefore thoroughly revised the affected sections:

*	The immune cell infiltration analysis (Section 3.6) and the corresponding discussion have been re-written to interpret the correlations based on the accurate expression patterns of the core targets.

*	The proposed Adverse Outcome Pathway (AOP) framework (Section 3.9) and the theoretical discussion in Sections 4.2 and 4.3 have been fundamentally re-evaluated and reconstructed to align with the verified expression data, ensuring the proposed mechanism is logically sound and consistent with our findings.

Once again, we would like to express our sincere gratitude for your valuable time and professional guidance. We believe that the revisions made in response to your comments have greatly improved the quality, rigor, and completeness of our manuscript. We hope that the revised version meets PLOS ONE’s publication standards. Please do not hesitate to contact us if you have any further questions or require additional revisions.

Sincerely,

The Authors

**Response to Reviewer #4:**

**Dear Reviewer #4,**

We sincerely thank the reviewer for their thorough and constructive assessment of our manuscript. Their insightful comments have been invaluable in helping us improve the quality and clarity of our work. We have addressed each point raised in detail below.

**Comment 1:** Line 58: A reference is missing to support the statement regarding serum BaA levels in LUAD patients.

**Response:** We thank the reviewer for pointing out this oversight. Upon careful re-evaluation, we found that the statement regarding serum BaA levels in LUAD patients was based on indirect evidence and lacked a direct, robust reference. Therefore, we have removed this specific claim from the manuscript (Introduction, line 58) to ensure accuracy.

**Comment 2:** Figure 2B: The KEGG pathway enrichment includes “pathways in cancer” and multiple general cancer terms. Since this study focuses specifically on LUAD, the enrichment pattern appears overly broad, and it is unclear why no immune-related pathways are highlighted. The authors should clarify or refine the enrichment criteria.

**Response:** We appreciate the reviewer's insightful comment regarding the KEGG enrichment results. The appearance of broad terms like "Pathways in cancer" is a known characteristic of the KEGG database's hierarchical structure, where specific pathways (like the Chemokine signaling pathway, which is highly immune-related) are children of these broader parent terms. Our analysis did identify and highlight several immune-related pathways. Specifically, the "Chemokine signaling pathway" was one of the most significantly enriched pathways, and it plays a crucial role in immune cell recruitment and activation. Furthermore, the "Viral protein interaction with cytokine and cytokine receptor" pathway is also intimately linked to immune regulation. We have revised the text in Section 3.2 to better clarify this point and to explicitly emphasize the relevance of the identified immune-associated pathways, such as the chemokine pathway, to the LUAD immune microenvironment.

**Comment 3:** Figure 4: The authors used the GSE10072 dataset for expression validation. This microarray dataset is relatively small and outdated compared to the TCGA-LUAD RNA-seq cohort. To reduce bias and enhance reliability, the authors should validate their results using at least one larger RNA-seq dataset.

**Response:** We fully agree with the reviewer regarding the importance of robust validation. In response, we have significantly strengthened our validation approach by incorporating two additional independent LUAD transcriptomic datasets from the GEO database: GSE32863 (58 LUAD and 58 matched adjacent normal tissues) and GSE31210 (226 LUAD and 20 normal lung tissues). We also explicitly state the sample size of GSE10072 (58 LUAD and 49 normal tissues) in the revised manuscript. The three datasets were integrated, batch effects were removed, and quantile normalization was applied. The consistent differential expression of the five core targets (TNNC1, ABCC3, CRABP2, CXCL12, OLR1) across all three independent cohorts (p < 0.01), with AUC values ranging from 0.841 to 0.886, strongly confirms the stability and reliability of our core target identification. These updated analyses and results are now detailed in Section 3.4 and the corresponding figures.

**Comment 4:** Figure 5A: The text states that MMRN2 is enriched in the treatment group; however, in the figure, MMRN2 appears higher in the control group. Please verify and correct this discrepancy.

**Response:** We are grateful to the reviewer for their meticulous observation. Following a refinement of the BaA-related target screening criteria and the application of three machine learning methods, the final set of core targets has been revised to five, and MMRN2 is no longer among them. We have meticulously re-examined the expression data for the five definitive core targets in the updated figures (Fig. 4C, formerly Fig. 5A) and have corrected all textual descriptions in the manuscript (Sections 3.4 and 4.2) to accurately reflect the verified expression patterns for each specific gene.

**Comment 5:** Section 3.6: Figures are not labeled properly (e.g., subpanels A–D in Figure 6). Each panel should be clearly annotated and referenced in the text.

**Response:** We thank the reviewer for highlighting this lack of clarity. We have carefully reviewed and corrected all figure labels in Section 3.6 (and throughout the manuscript). Each subpanel (A, B, C, etc.) is now clearly annotated in the figures themselves and is properly referenced in the corresponding text to ensure ease of understanding for the reader.

**Comment 6:** Line 299: The manuscript claims that MMRN2 exhibits the most significant negative correlation with M0 macrophages, but Figure 6D does not support this. Please recheck the correlation analysis and adjust the text accordingly.

**Response:** We sincerely apologize for this error. As mentioned in our response to Comment #4, MMRN2 is not part of the final five core targets. Consequently, all statements regarding MMRN2's correlations have been removed from the manuscript. The immune infiltration analysis in Section 3.5 and the corresponding discussion in Section 4.3 now focus exclusively and accurately on the immunomodulatory patterns of the five definitive core targets: TNNC1, ABCC3, CRABP2, CXCL12, and OLR1.

**Comment 7:** Figure 6B: The stated differences between control and treatment groups for CD4-negative T cells, activated NK cells, and M1 macrophages do not appear statistically significant. This should be corrected or clarified in the figure legend.

**Response:** We thank the reviewer for this precise observation. We have re-analyzed the immune infiltration data and corrected the figure (now Fig. 5B) and the corresponding text in Section 3.5.

**Comment 8:** Figure 7: Docking scores around –6 kcal/mol indicate moderate, not strong, binding affinity. The authors should revise the text to reflect this.

**Response:** We thank the reviewer for this correction. We have revised the text in Section 3.6 to accurately describe the binding affinities.

**Comment 9:** Figure 8 (RMSF plot): The protein appears to display widespread flexibility across residues, with no clear stable core region. The authors should clarify whether this reflects true intrinsic flexibility of the protein or potential artifacts from suboptimal normalization or simulation parameters.

**Response:** We thank the reviewer for their comment on the RMSF profile. After optimizing the screening criteria for BaA-related targets, we re-screened using three machine learning methods and identified five core targets. The selected compound for docking was BaA-CRABP2, with a molecular docking binding energy of-8.4 kcal/mol. We further conducted kinetic simulations on it.The observed fluctuations in this simulation were not artifacts but reflected the intrinsic dynamic properties of the CRABP2 protein. As characteristic of proteins from the intracellular lipid-binding protein family, CRABP2 features a stable β-barrel core, which exhibits low RMSF values (as seen in the stable baseline of the plot), connected by flexible loop regions that display higher RMSF. This pattern of 'stable elements connected by flexible linkers' is a hallmark of this protein family and is crucial for its function. The high flexibility in the loops may facilitate ligand entry, binding, and release. Therefore, the RMSF profile confirms that the simulation successfully captured the natural dynamics of the protein, with a stable core and functionally important flexible regions, rather than indicating systemic instability

**General Comment 1:** Figure legends are overly simplistic and lack methodological detail. Please describe normalization methods, parameter settings, and statistical tests for each analysis.

**Response:** We thank the reviewer for this important suggestion. We have comprehensively revised all figure legends throughout the manuscript. Each legend now includes essential methodological details such as normalization procedures, key parameter settings, and the specific statistical tests employed for the analysis presented in the figure.

**General Comment 2:** Several key findings are not adequately discussed in the Results or Discussion sections.

**Response:** We have carefully reviewed the Results and Discussion sections to ensure that all key findings, particularly those related to the five core targets, their expression validation across multiple datasets, their immunomodulatory roles, and the computational binding analyses, are now thoroughly discussed and interpreted in the context of existing literature.

**General Comment 3:** The Discussion section is overly repetitive. In particular, lines 542–561 are redundant and somewhat unrelated to the study’s core topic.

**Response:** We sincerely thank the reviewer for this feedback. We have thoroughly revised the entire Discussion section to eliminate redundancy and to sharpen the focus on the core findings and implications of our study. As suggested, the redundant content previously found in lines 542-561 has been removed. The discussion now flows more logically and concisely.

Once again, we would like to express our sincere gratitude for your valuable time and professional guidance. We believe that the revisions made in response to your comments have greatly improved the quality, rigor, and completeness of our manuscript. We hope that the revised version meets PLOS ONE’s publication standards. Please do not hesitate to contact us if you have any further questions or require additional revisions.

Sincerely,

The Authors

**Response to Reviewer #5:**

**Dear Reviewer #5,**

We sincerely thank the reviewer for their positive assessment of the scientific validity and relevance of our work, as well as for their insightful and constructive comments. Their suggestions have been invaluable in strengthening the methodological clarity and robustness of our study. We have addressed each point in detail below.

**Comment 1:** Please clarify the workflow for identifying BaA targets. Specify how many targets were obtained from each database and state whether these were direct, high-confidence targets for BaA or included predicted/inferred associations.

**Response:** We thank the reviewer for this suggestion to improve the clarity of our target identification workflow. We have now provided a more detailed description in Section 2.1. Specifically, potential human targets for BaA were collected from the ChEMBL (432 targets), SwissTargetPrediction (4 targets, with Probability > 0), and Comparative Toxicogenomics Database (CTD, 12 targets, with Reference Count > 1). To ensure high confidence and exclude pan-assay interference compounds (PAINS) and low-confidence targets, we applied stringent filters: only targets from SwissTargetPrediction with a probability score greater than 0 and from CTD with a reference count greater than 1 were retained. After merging and removing duplicates, a final set of 444 unique targets constituted our high-confidence BaA target library. This refined list, comprising a mix of direct and high-confidence predicted targets, was used for all subsequent analyses. The specific numbers from each database are now clearly stated in Section 3.1 and Figure 1A.

**Comment 2:** The reported number of BaA-related targets (1,943) seems unusually high for a single pollutant. Please confirm the composition of this list and verify that it does not contain unreliable, indirect associations that could affect the robustness of the subsequent 651 intersection targets and enrichment analysis.

**Response:** We appreciate the reviewer's vigilance regarding the initial target number. We sincerely apologize for the typographical error and any confusion it caused. The correct, final number of high-confidence BaA-related targets, after applying the stringent filters described above, is 444, not 1,943. Consequently, the number of intersection targets between BaA and LUAD is 248, not 651. We have carefully corrected these numbers throughout the manuscript (Sections 2.1, 3.1, and Figure 1) and verified that the subsequent functional enrichment analysis is based on this robust set of 248 intersection targets.

**Comment 3:** The machine learning methodology requires more detail to prove robustness. Please specify the size and class balance (normal vs. LUAD) of the training dataset, state if feature scaling was applied, and justify the use of 3-fold cross-validation. Especially, the diagnostic performance of the 7-gene signature must be validated in a fully independent cohort (e.g., TCGA-LUAD), not just within the same GSE10072 dataset.

**Response:** We thank the reviewer for these critical suggestions to enhance the robustness of our machine learning approach. We have substantially revised Section 2.4 to include the requested details. The core targets were identified from shared targets between BaA and LUAD. Subsequently, we validated these core targets by incorporating two additional independent cohorts (GSE32863 and GSE31210) along with the original cohort GSE10072, thereby integrating three GEO datasets.

**Comment 4:** The immune infiltration analysis needs to address its limitations. The use of the small GSE10072 microarray dataset (n=58) can lead to unstable results. Please state if batch correction was performed and ensure the biological interpretation of immune correlations is conservative.

**Response:** We agree with the reviewer regarding the limitations of using a single, small dataset for immune analysis. To address this, we have significantly strengthened our approach. The immune infiltration analysis is now based on the integrated and batch-corrected dataset (GSE10072, GSE32863, GSE31210). This larger sample size provides more stable and reliable estimates of immune cell abundances. Furthermore, in Section 3.5 and the Discussion (4.3), we have adopted a more conservative tone in interpreting the correlations between core targets and immune cells, presenting them as potential associations within the tumor microenvironment that warrant further experimental investigation.

**Comment 5:** The molecular docking and dynamics simulations need more rationale. For proteins not known as classical ligand-binders (e.g., FOXF1, CD34), explain how the binding pockets were defined. Justify why only the BaA-MMRN2 complex was selected for molecular dynamics simulation.

**Response:** We thank the reviewer for raising these important methodological points.

Binding Pocket Definition: We acknowledge the challenge with non-classical ligand-binding proteins. In our revised workflow, as clarified in Section 2.7, for proteins without a known, well-defined active site, the grid box for docking was centered on the largest putative binding cavity identified through computational cavity detection using AutoDock Tools.

Selection for MD Simulation: The primary criterion for selecting a complex for MD simulations was the strength of the binding affinity (the most negative Vina score) from the docking results, as this represents the most stable predicted interaction. In the revised manuscript, the complex selected for detailed MD simulation analysis is the one with the strongest binding affinity, which is the **BaA-CRABP2** complex. This rationale is now explicitly stated in Section 2.8.

**Comment 6:** The constructed Adverse Outcome Pathway (AOP) is currently too speculative. It should be explicitly framed as a putative or hypothetical AOP, with a clear statement that the Key Event Relationships require direct experimental validation. A limitations paragraph acknowledging this is necessary.

**Response:** We fully agree with the reviewer. We have reframed our AOP throughout the manuscript as a "AOP hypothesis". We have also added a clear statement in Section 3.8 emphasizing that the proposed Key Event Relationships are speculative and require direct experimental validation. Furthermore, we have strengthened the "Limitations and Future Perspectives" section (Section 4.6) to explicitly acknowledge the hypothetical nature of the AOP and the critical need for future wet-lab experiments to validate the proposed mechanistic links.

Once again, we would like to express our sincere gratitude for your valuable time and professional guidance. We believe that the revisions made in response to your comments have greatly improved the quality, rigor, and completeness of our manuscript. We hope that the revised version meets PLOS ONE’s publication standards. Please do not hesitate to contact us if you have any further questions or require additional revisions.

Sincerely,

The Authors
