# Supplementary material for: Exploring the toxicological mechanisms of Benzo[a]anthracene (BaA) exposure in lung adenocarcinoma (LUAD) via network toxicology, machine learning, and multi-dimensional bioinformatics analysis
Source: PLoS One. 2026 Feb 11;21(2):e0340116. doi: 10.1371/journal.pone.0340116 (PMC12893569; doi:10.1371/journal.pone.0340116)
Supplement: Attachment — Submitted filename: Response_to_Reviewers_auresp_2.docx [file pone.0340116.s006.docx]

**Response to Reviewers**

Dear Editor and Reviewers,

Thank you for the opportunity to revise our manuscript entitled "Exploring the toxicological mechanisms of Benzo[a]anthracene (BaA) exposure in lung adenocarcinoma via network toxicology, machine learning, and multi-dimensional bioinformatics analysis" (Manuscript ID: PONE-D-25-55154). We sincerely appreciate the valuable comments and suggestions from all reviewers, which have helped us significantly improve the quality of our work.

We have carefully addressed all points raised in the review process. Below, we provide a point-by-point response to each comment. All corresponding changes have been incorporated into the revised manuscript using the "Track Changes" function.

We believe that the criticisms have been addressed effectively and that the revisions have substantially strengthened the manuscript. We are grateful for the guidance provided and hope that the revised version now meets the high publication standards of PLOS ONE.

Thank you again for considering our work. We look forward to hearing from you.

Sincerely,

Zhiyao Shi, Xixing Wang
On behalf of all co-authors

**Response to Editorial Requirements：**

Dear Dr. Jiafu Li and the Editorial Team,

On behalf of all authors, I would like to extend our sincere gratitude to you and the editorial team for your time and consideration in evaluating our manuscript entitled “Exploring the toxicological mechanisms of Benzo[a]anthracene (BaA) exposure in lung adenocarcinoma (LUAD) via network toxicology, machine learning, and multi-dimensional bioinformatics analysis” (Submission ID: PONE-D-25-55154R1).

We are deeply appreciative of the opportunity to revise our manuscript and for the constructive feedback provided by the reviewers, which has significantly improved the quality of our work. We have carefully considered all comments and have made comprehensive revisions to address each point raised. Our detailed point-by-point responses are included in the “Response to Reviewers” file accompanying this submission.

Regarding the editorial suggestion on sharing laboratory protocols via protocols.io, we would like to provide the following clarification:

We sincerely thank the editor and reviewers for this constructive suggestion regarding enhancing reproducibility. As our study is primarily a bioinformatics and computational analysis based on publicly available datasets (e.g., GEO, TCGA) and does not involve original laboratory experiments, there are no wet-lab protocols to deposit at this stage. All analytical workflows, scripts, and parameter files have been made publicly available in our Zenodo repository (DOI: [10.5281/zenodo.17747122](https://doi.org/10.5281/zenodo.17747122)), as stated in the Data Availability Statement. Should we conduct experimental validations in future studies, we will certainly utilize protocols.io to share detailed methodologies.

We have prepared the following documents for your review:

1. Response to Reviewers: A detailed point-by-point response to all reviewer comments.

2. Revised Manuscript with Track Changes: A version highlighting all modifications.

3. Manuscript (Clean Version): The final, unmarked version of the revised manuscript.

We believe that our revisions have strengthened the manuscript and hope it is now suitable for publication in PLOS ONE.

Furthermore, as the first author is scheduled to graduate next year, the acceptance of this manuscript in 2025 is crucial for meeting degree requirements. This study systematically integrates network toxicology, machine learning, and multi-dimensional bioinformatics approaches to elucidate the toxicological mechanisms of benzo[a]anthracene in lung adenocarcinoma and constructs an adverse outcome pathway (AOP)-based theoretical framework, providing novel methodologies and foundational insights for environmental pollutant risk assessment. We believe this research will contribute meaningfully to the fields of environmental toxicology and lung cancer prevention.

Considering the importance of this publication for our academic and professional advancement, we kindly request your consideration in expediting the remaining steps of the review process, if possible. We fully understand that the peer-review process requires careful evaluation to maintain the journal's quality and integrity. However, any assistance in facilitating the subsequent stages would be greatly appreciated.

Thank you once again for your consideration and support. We look forward to hearing from you regarding our manuscript at your earliest convenience. Should you require any additional information or have any questions, please do not hesitate to contact us.

Sincerely,

Zhiyao Shi, Xixing Wang
Department of Oncology

Shanxi Provincial Hospital of Traditional Chinese Medicine

Taiyuan, Shanxi, China

Email: 18234069215@163.com，wangxx315@163.com

On behalf of all co-authors

**Response to Reviewer #1:**

**Dear Reviewer #1,**

We sincerely appreciate your meticulous review and valuable insights on our manuscript. Your professional comments have provided crucial guidance for improving the quality and rigor of our study, and we fully agree with all the revision suggestions you proposed. We have carefully addressed and completed the necessary revisions to this feedback. Below is our detailed response to all your comments:

**Comment 1:** Most of the required supplementary experiments or analyses have been completed; however, it is recommended that a major revision be conducted again. First and foremost, I fully understand the experimental constraints faced by the author team, such as being unable to perform in vivo or in vitro experiments. Nevertheless, at the very least, the number of public transcriptomic datasets should be increased, instead of being limited to a single dataset. If the authors are even unable to expand the number of public datasets used for analysis, I recommend that the manuscript be rejected.

**Response:** We sincerely appreciate your thorough review of our manuscript and your constructive feedback. We fully understand the importance of expanding the dataset to strengthen the robustness of our findings. Regarding your primary concern about the limited number of public transcriptomic datasets, we would like to clarify that in the current version, we have already utilized three independent GEO datasets (GSE10072, GSE32863, and GSE31210) for our core analysis and validation. Furthermore, we utilized the UALCAN platform to validate the expression patterns of identified key targets in an independent TCGA-LUAD cohort, ensuring the reproducibility and cross-cohort reliability of our findings.

We acknowledge that increasing the number of datasets could further reinforce the statistical power of the study. As graduate students nearing the completion of our degrees, this study represents a critical component of our graduation requirements, and we have strived to conduct comprehensive bioinformatics analyses within the scope of our capabilities. We kindly request your consideration regarding the current validation framework, which already integrates multiple independent cohorts.

Once again, we would like to express our sincere gratitude for your valuable time and professional guidance. Thank you for your understanding and for the opportunity to improve our work. We hope that the revised version meets PLOS ONE’s publication standards.

Sincerely,

The Authors

**Response to Reviewer #2:**

**Dear Reviewer #2,**

We are sincerely grateful to you for your time and insightful review of our manuscript. Your constructive feedback during the previous round has been instrumental in improving both the clarity and scientific rigor of our work. We are delighted to know that you find our responses satisfactory and that the revised version now meets the standards for publication in your esteemed journal. Thank you once again for your guidance and for the valuable role you have played in refining this study.

Sincerely,

The Authors

**Response to Reviewer #3:**

**Dear Reviewer #3,**

We extend our deepest gratitude for your continued engagement and thoughtful evaluation of our manuscript. Your comments during the initial review were exceptionally helpful in shaping the direction of our revisions, and we are truly humbled by your acknowledgment that we have adequately addressed your concerns. It is an honor to receive such positive feedback from a scholar of your standing. Thank you for your patience, expertise, and invaluable contributions to improving this work.

Sincerely,

The Authors

**Response to Reviewer #4:**

**Dear Reviewer #4,**

Thank you sincerely for your thorough review and valuable suggestions on our revised manuscript. We greatly appreciate your recognition of the significant improvements made and your constructive feedback on the remaining issues. We have carefully addressed each of your concerns, and the detailed revisions are outlined below:

**Comment 1:** Several figures remain low in resolution, and the font styles and sizes are not consistent across panels. These should be standardized to ensure clarity and readability.

**Response:** We thank you for this important observation regarding figure quality and consistency. We have regenerated all relevant figures at high resolution and have systematically standardized the font style and size across all panels within each figure and throughout the figure set. We believe these adjustments significantly improve the visual clarity and professional presentation of the data.

**Comment 2:** Some legends do not provide sufficient detail for the reader to understand the experiment without referring back to the main text. For example, the legends for Figures 4A and 4B are too simplified and should be expanded to include the axis information and relevant statistical information.

**Response:** We have expanded the legends for Figures 4A and 4B, as well as other potentially insufficient legends, to include axis definitions, statistical criteria, and sample information, enabling readers to understand the experiments without referring to the main text:

Figure 4A (Volcano plot): Revised legend: “Fig. 4A. Volcano plot of differentially expressed genes (DEGs) from integrated GEO datasets (GSE10072, GSE32863, GSE31210). The x-axis represents log₂ fold change (log₂FC) of gene expression (LUAD vs. normal samples), and the y-axis represents -log₁₀ (adjusted p-value). Red dots indicate upregulated DEGs (log₂|FC| > 1, adjusted p < 0.05), blue dots indicate downregulated DEGs (log₂|FC| > 1, adjusted p < 0.05), and gray dots indicate non-significantly differentially expressed genes.”

Figure 4B (Heatmap): Revised legend: “Fig. 4B. Heatmap of the top 50 DEGs from integrated GEO datasets (GSE10072, GSE32863, GSE31210). Rows represent individual genes, columns represent samples (blue = normal samples, red = LUAD samples). The color gradient (blue to red) indicates normalized gene expression levels (blue = low expression, red = high expression), with values standardized by z-score. DEGs were screened using the criteria: log₂|FC| > 1 and adjusted p < 0.05.”

Other figures (e.g., Figures 2A, 2B, 5B) have also been supplemented with similar details, including data sources, statistical standards, and the meaning of color/bubble size.

**Comment 3:** In several instances, the authors merely describe the figure without summarizing the key conclusion or interpretation. For example, Line 551 should include a brief concluding sentence that highlights the main takeaway from the results shown. This issue applies to other result subsections as well.

**Response:** We have added concise concluding sentences to result subsections where only figure descriptions were provided, highlighting the core takeaways of each section:

Line 551 (Section 3.3: Identification of core targets via machine learning algorithms): Added: “The triple-algorithm screening (LASSO, SVM-RFE, and Random Forest) ensures high confidence in these five core targets, which are likely key mediators linking BaA exposure to LUAD progression.”

Section 3.1 (Network toxicology analysis): Added: “These 248 intersection targets provide a molecular basis for understanding the multi-target regulatory mechanism of BaA-induced LUAD.”

Section 3.2 (Functional enrichment analysis): Added: “The enriched pathways collectively suggest that BaA may promote LUAD by disrupting immune regulation, activating oncogenic signaling, and interfering with cytokine receptor interactions.”

Section 3.5 (Immune infiltration analysis): Added: “These findings reveal a dual immunomodulatory pattern of core targets, which may reshape the tumor immune microenvironment to facilitate LUAD development.”

Similar concluding sentences have been added to Sections 3.4, 3.6, 3.7, 3.8, and 3.9 to summarize the biological significance of the results.

**Comment 4:** Lines 608–609: The phrase “lower binding affinity value” is confusing in this context. If the authors are referring to binding free energy from docking analyses, a clearer phrasing should be “lower binding free energy value.”

**Response:** We have clarified the confusing phrasing related to binding affinity:

Original sentence: “Lower binding affinity values (i.e., more negative) correspond to stronger intermolecular forces and greater conformational stability between the ligand and the target protein.”

Revised sentence: “Lower binding free energy values (i.e., more negative) from molecular docking correspond to stronger intermolecular forces and greater conformational stability between BaA (ligand) and the target proteins.”

This revision explicitly specifies that the value refers to “binding free energy from docking analyses” and clarifies the ligand-target relationship, eliminating ambiguity.

All revisions have been carefully implemented to address your concerns while maintaining the scientific rigor and consistency of the manuscript. Once again, we would like to express our sincere gratitude for your valuable time and professional guidance. We believe that the revisions made in response to your comments have greatly improved the quality, rigor, and completeness of our manuscript. We hope that the revised version meets PLOS ONE’s publication standards. Please do not hesitate to contact us if you have any further questions or require additional revisions.

Sincerely,

The Authors

**Response to Reviewer #5:**

**Dear Reviewer #5,**

We sincerely thank the reviewer for their positive assessment of the scientific validity and relevance of our work, as well as for their insightful and constructive comments. Their suggestions have been invaluable in strengthening the methodological clarity and robustness of our study. We have addressed each point in detail below.

**Comment 1:** Removing all residual text referring to the former seven-gene set and ensuring that only the finalized five-gene signature is described throughout (e.g., replace "seven" with "five" in line 443).

**Response:** We have carefully reviewed the entire manuscript and systematically replaced all mentions of the earlier "seven-gene set" with the final "five-gene signature." The text in line 443 has been corrected. The text now consistently refers only to the validated five-gene model in all sections, including the Introduction, Methods, Results, and Discussion.

**Comment 2:** Unifying terminology used in section headings, figure legends, and the main text.

**Response:** Regarding the unification of terminology: We have thoroughly standardized the terminology used in section headings, figure legends, and the main text. Key revisions include: (1) Consistently using "core targets" instead of "key targets" to avoid ambiguity; (2) Unifying the description of intersection targets as "intersection targets for BaA-induced LUAD" to ensure consistency across sections; (3) Standardizing abbreviations (e.g., first defining "molecular dynamics (MD)" and "adverse outcome pathway (AOP)" before using their abbreviations); (4) Aligning terminology in figure legends with the main text; (5) Unifying database and protein names according to field conventions (e.g., "Swiss Target Prediction" instead of "Swiss," and standardizing the capitalization of protein names such as "Cellular retinoic acid binding protein 2 (CRABP2)").

We have conducted a thorough review of all terminology across section headings, figure legends, table captions, and the main body of the text. Any inconsistencies in naming conventions for key variables, model components, or analytical methods have been harmonized to ensure clarity and consistency throughout the manuscript.

Once again, we would like to express our sincere gratitude for your valuable time and professional guidance. We believe that the revisions made in response to your comments have greatly improved the quality, rigor, and completeness of our manuscript. We hope that the revised version meets PLOS ONE’s publication standards. Please do not hesitate to contact us if you have any further questions or require additional revisions.

Sincerely,

The Authors
